# A Neonate With Annular Cutaneous Lesions

**DOI:** 10.7759/cureus.9138

**Published:** 2020-07-11

**Authors:** Vivian Vega, Mayela Duque, Data Don-Pedro, Sabita Bhatta

**Affiliations:** 1 Pediatrics, Woodhull Medical Center, Brooklyn, USA

**Keywords:** lupus, pediatric dermatology, neonatology, pediatric rheumatology, pediatrics and neonatology

## Abstract

Neonatal lupus erythematosus is a rare disorder with a wide spectrum of clinical presentations. The disease can affect several systems, including the skin, heart, liver, and bone marrow. Thus, a recognition of its different presentations is vital for making a diagnosis. We describe the case of a neonate diagnosed with neonatal lupus, presenting with an acute onset of erythematous skin lesions as the sole manifestation. The patient’s mother was healthy with no known medical history or relevant family history

## Introduction

Neonatal lupus erythematosus (NLE) is a rare condition that develops from the passive maternal transfer of antibodies across the placenta to the fetus. It can manifest with a wide range of manifestations [1,2]. We describe the case of a neonate diagnosed with NLE, presenting with an acute onset of erythematous skin lesions as the sole manifestation of the disease. The patient’s mother was healthy with no known medical history or relevant family history. 

## Case presentation

The patient is a male infant who was born full term at 39 weeks via spontaneous vaginal birth. His birth history was otherwise unremarkable. The mother is a 26-year-old female, gravida 3 para 2 with no reported significant past medical history or family history. The mother had an unremarkable pregnancy and reports having completed all of her prenatal visits. 

The patient was admitted to our institution at 25 days of age when he developed an acute onset of skin lesions on the face, upper neck, and abdomen. The mother reports the skin lesions developed three days after the patient's first exposure to direct sunlight when she took him for a walk that lasted about 35 minutes.

**Figure 1:**
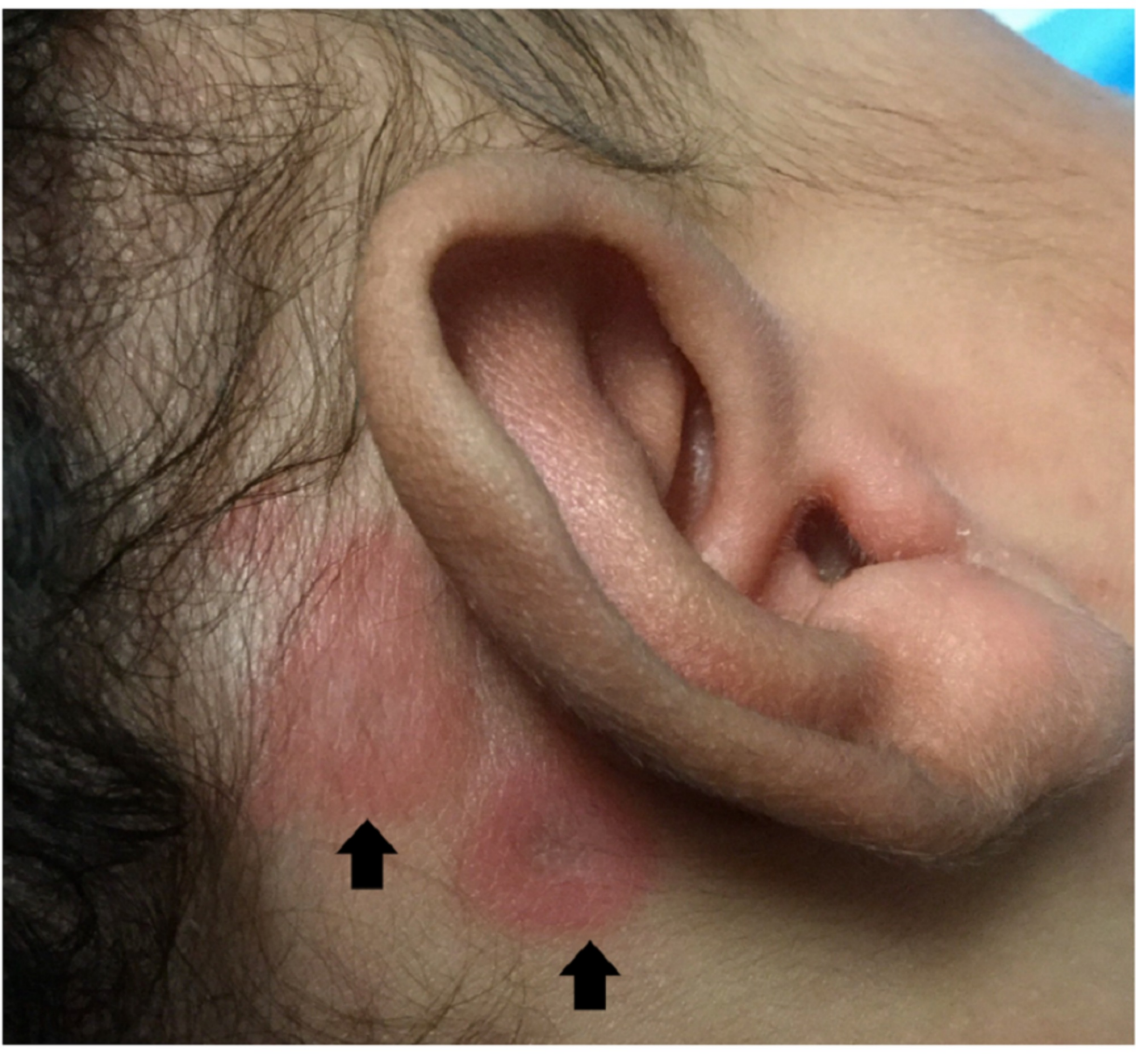
Annular erythematous lesions with central atrophy and raised borders observed behind the ear (black arrows).

**Figure 2:**
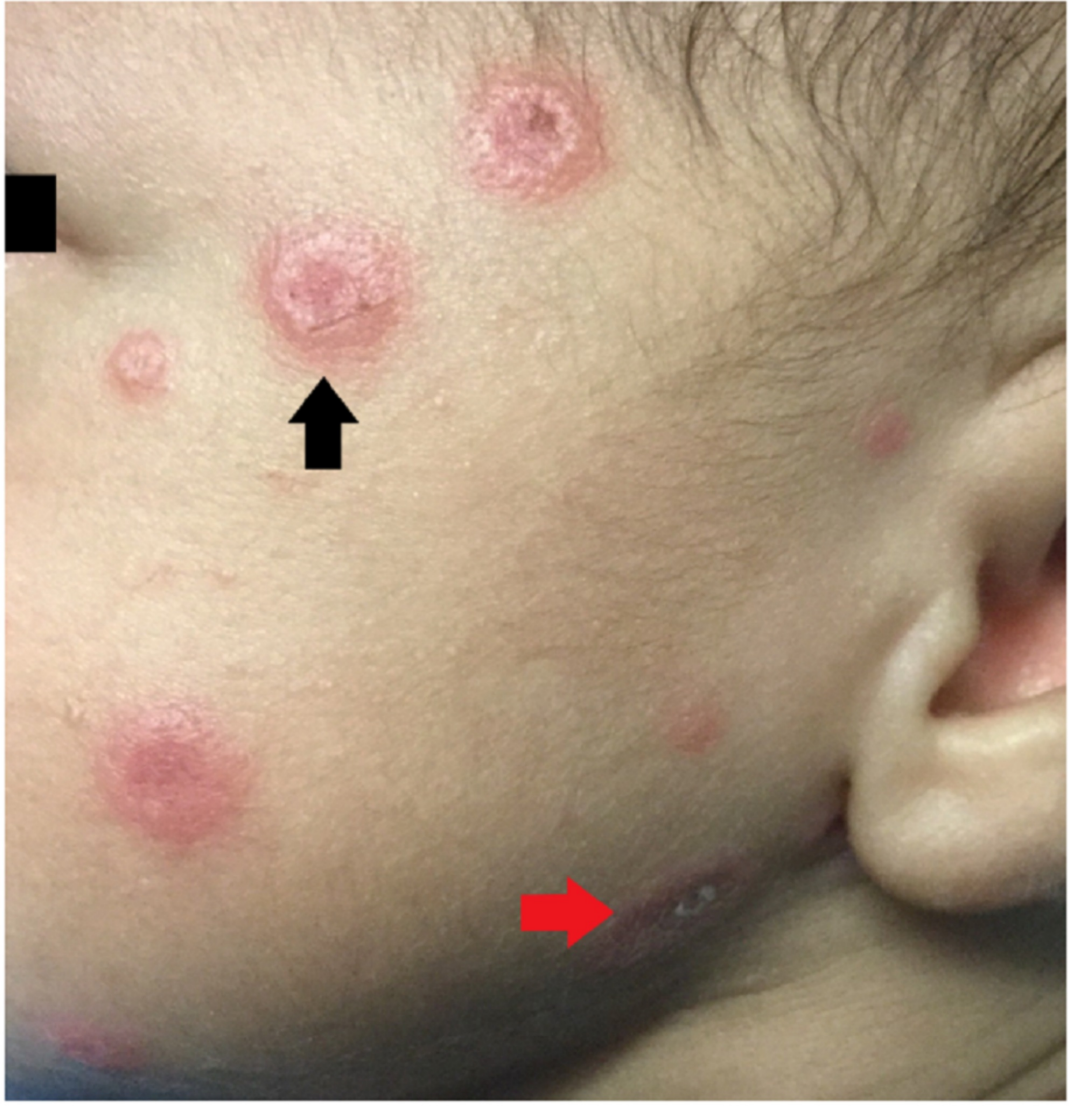
An example of an annular erythematous plaque with central atrophy and raised borders (black arrow) and an erythematous desquamating lesion (red arrow) observed in the patient's face.

**Figure 3:**
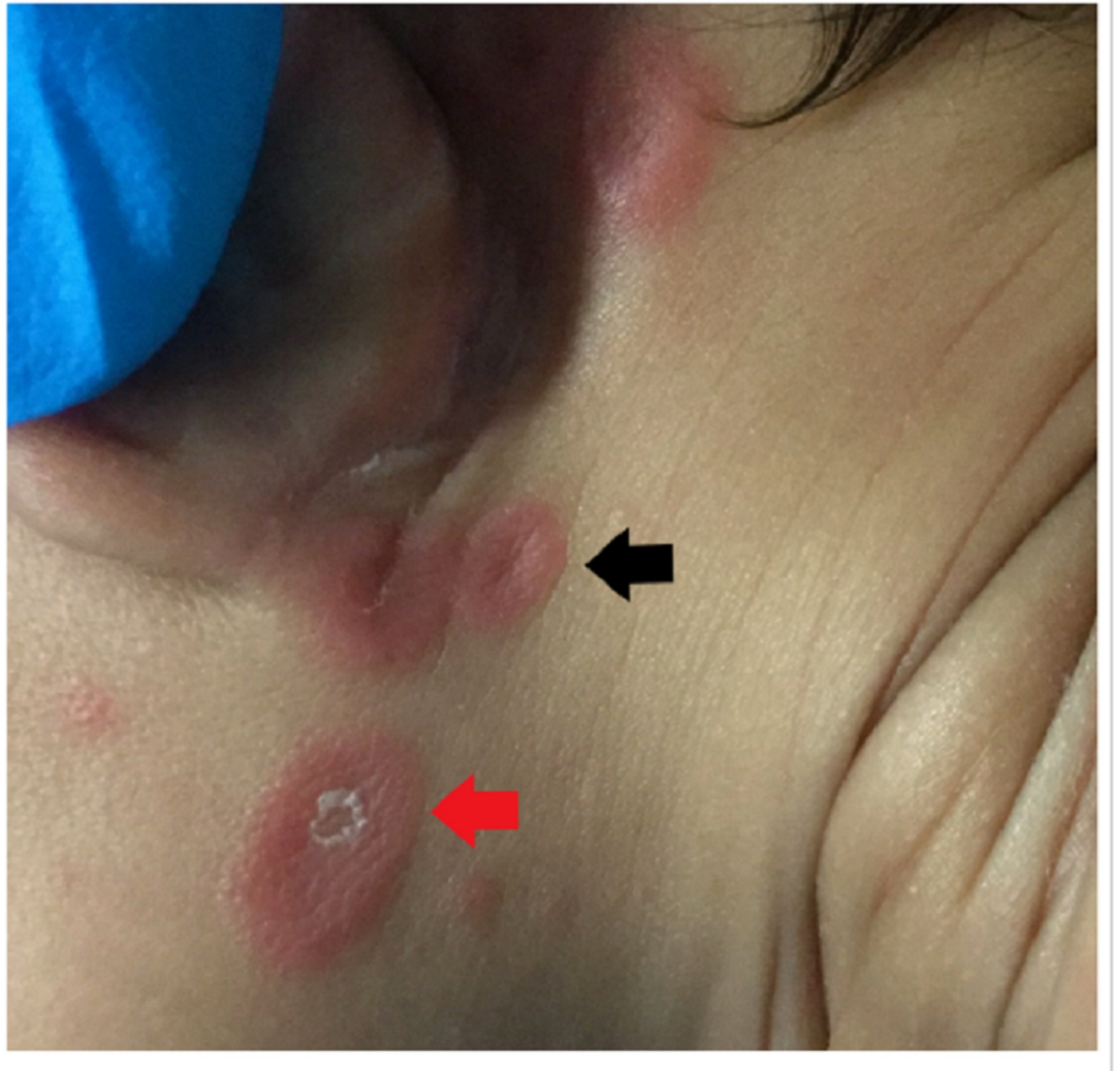
The patient's neck area showing annular erythematous plaques with central atrophy and raised borders (black arrow) and an erythematous desquamating plaque (red arrow).

On physical exam, the patient was noted to be awake and active, the skin had multiple lesions described as circumscribed plaques of different sizes, erythematous, annular with central atrophy, and raised borders. Some of the plaques presented skin desquamation. The lesions were located over the post-auricular area, face, and scalp (Figures 1-3). The lesions were non-pruritic and non-migrating, and did not involve the peripheral areas of the body.

The patient was otherwise doing well, afebrile, breastfed exclusively, and growing well. On admission, his baseline labs, including complete blood count, basic metabolic panel, hepatic panel, and urine analysis, were unremarkable. An electrocardiogram (ECG) was performed and did not show evidence of any conduction abnormalities.

To confirm the diagnosis, the child and mother were tested for antinuclear antibodies (ANA) and anti-Ro/anti-La. The child had positive anti-Ro/anti-LA and ANA positive in high titers with speckled pattern, and the mother tested positive for anti-Ro/anti-La but at the time was healthy and did not have any signs or symptoms of autoimmune disease 

The dermatology team was consulted and recommended topical triamcinolone to hasten the resolution of the skin lesions. 

After starting the treatment, the lesions were found to be improving. The patient was discharged on hospital day 3 and scheduled to be seen by cardiology as an outpatient. 

## Discussion

NLE is a rare condition and often present a diagnostic challenge. The differential diagnoses can range from benign self-resolving conditions to life-threatening systemic illnesses.

The most common manifestation of NLE is cutaneous involvement, which occurs in about 40% of patients [1,2]. There are multiple conditions with similar dermatologic findings, such as atypical erythema multiforme, erythema annulare, urticaria, tinea corporis, familial annular erythema, and erythema gyratum athopicans, and should be considered as differential diagnosis [3,4].

These conditions can be differentiated by the timing of onset, the morphology, and distribution of the rash along with associated symptoms and history. 

The distribution of the lesions on our patient is typical for NLE which usually affects sun-exposed areas, and the lesions are usually exacerbated by ultraviolet light exposure [5]. The timing of the lesions can be present at birth but typically tend to occur between four to six weeks after birth, as in this patient. Other manifestations can include cardiac abnormalities, such as varying degrees of heart block, cardiomyopathy, or congenital abnormalities. Hepatobiliary abnormalities like liver failure, transaminase elevations, and hyperbilirubinemia are observed in about 10% of patients. Hematologic manifestations like thrombocytopenia and neutropenia have been reported as well [1-6]. 

NLE results from transplacental transfer of maternal autoantibodies to the fetus. The autoantibodies that are usually involved are anti-Ro, anti-La, or anti-RNP. In patients with cutaneous manifestations or with atrioventricular block, NLE is diagnosed when the mother has positive anti-Ro/anti-La antibodies. In this case, both the patient and mother tested positive for the antibodies [7,8]. 

The mother of the child may have an autoimmune disorder such as lupus, Sjogren’s syndrome, rheumatoid arthritis, or other connective tissue disorders in about 75% of the cases, but it is also important to note that in some cases as in this case, the child's mother can be asymptomatic and healthy at the time of diagnosis [2,7,8]. 

Once the diagnosis is established, these neonates should be screened for conduction abnormalities with an ECG in all neonates. Approximately 25% of patients present cardiac involvement. Follow up with an ECG and an echocardiogram is recommended for those with first degree and second degree. It is important to note that up to 2% of infants develop atrioventricular blocks within the first month [9]. 

Avoidance of sun exposure is the mainstay of management for cutaneous manifestations. The management of NLE is primarily expectant, but complete resolution of the skin lesions takes within six to nine months [5,6,10]. Topical steroids have been used to expedite the resolution of the lesions.

The counseling for parents of children diagnosed with NLE is utterly important. Children who have had NLE are at increased risk of autoimmune disease later in life, and the degree of the risk is still not clear as most data come from small case series, but it has been estimated to be around 2% [11,12]. The parents should be aware that subsequent pregnancies can be affected as well, and it has been reported that about 20% of subsequent pregnancies can be affected by NLE [11,12]. 

## Conclusions

The presence of annular erythematous skin lesions in a neonate has a wide array of differentials. This case highlights the classic skin findings of NLE. A thorough history and physical exam are key for diagnosing this condition, being that skin involvement is the most common manifestation. Testing of the child and mother with ANA and anti-Ro/anti-La antibodies should be performed to confirm the diagnosis.

This patient did not present with any cardiac conduction abnormalities; nevertheless, all patients with a confirmed diagnosis should obtain an ECG and have cardiology follow-up as outpatients. The patient’s skin lesions improved after five days with the avoidance of sun exposure, and the use of topical steroids in the affected area. 
